# Intra-Tumoral Pharmacokinetics of Pazopanib in Combination with Radiotherapy in Patients with Non-Metastatic Soft-Tissue Sarcoma

**DOI:** 10.3390/cancers13225780

**Published:** 2021-11-18

**Authors:** Laura Molenaar-Kuijsten, Milan van Meekeren, Remy B. Verheijen, Judith V. M. G. Bovée, Marta Fiocco, Bas Thijssen, Hilde Rosing, Alwin D. R. Huitema, Aisha B. Miah, Hans Gelderblom, Rick L. M. Haas, Neeltje Steeghs

**Affiliations:** 1 Department of Pharmacy & Pharmacology, The Netherlands Cancer Institute—Antoni van Leeuwenhoek, Plesmanlaan 121, 1066 CX Amsterdam, The Netherlands; l.kuijsten@nki.nl (L.M.-K.); r.verheijen@nki.nl (R.B.V.); bas.thijssen@nki.nl (B.T.); h.rosing@nki.nl (H.R.); a.huitema@nki.nl (A.D.R.H.); 2 Department of Medical Oncology, Leiden University Medical Center, Albinusdreef 2, 2333 ZA Leiden, The Netherlands; M.van_Meekeren@lumc.nl (M.v.M.); a.j.gelderblom@lumc.nl (H.G.); 3 Department of Pathology, Leiden University Medical Center, Albinusdreef 2, 2333 ZA Leiden, The Netherlands; J.V.M.G.Bovee@lumc.nl; 4 Mathematical Institute Leiden University, Niels Bohrweg 1, 2333 CA Leiden, The Netherlands; m.fiocco@math.leidenuniv.nl; 5 Department of Biomedical Data Science, Section Medical Statistics, Leiden University Medical Center, Albinusdreef 2, 2333 ZA Leiden, The Netherlands; 6 Department of Clinical Pharmacy, University Medical Center Utrecht, Utrecht University, Heidelberglaan 100, 3584 CX Utrecht, The Netherlands; 7 Department of Pharmacology, Princess Máxima Center for Pediatric Oncology, Heidelberglaan 25, 3584 EA Utrecht, The Netherlands; 8 Department of Clinical Oncology, The Royal Marsden Hospital and The Institute of Cancer Research, 15 Cotswold Rd, London SM2 5NG, UK; Aisha.Miah@rmh.nhs.uk; 9 Department of Radiotherapy, The Netherlands Cancer Institute—Antoni van Leeuwenhoek, Plesmanlaan 121, 1066 CX Amsterdam, The Netherlands; r.haas@nki.nl; 10 Department of Radiotherapy, Leiden University Medical Centre, Albinusdreef 2, 2333 ZA Leiden, The Netherlands; 11 Department of Medical Oncology and Clinical Pharmacology, The Netherlands Cancer Institute—Antoni van Leeuwenhoek, Plesmanlaan 121, 1066 CX Amsterdam, The Netherlands

**Keywords:** intra-tumoral drug concentration, neoadjuvant treatment, pharmacokinetics, tyrosine kinase inhibitor, pazopanib, soft tissue sarcoma

## Abstract

**Simple Summary:**

Pazopanib plasma levels have been associated with treatment efficacy. Since pazopanib targets receptors present on cells in the vicinity of the tumor and on tumor cells themselves, measurement of pazopanib concentrations in tumor tissue might be an even better prognostic biomarker than plasma levels. The aim of our study was to quantify pazopanib concentrations in tumor tissue, correlate this with plasma concentrations, and assess whether this is a better biomarker for efficacy. A modest correlation was found between pazopanib tumor concentrations and plasma concentrations. Additionally, no correlation was found between pazopanib tumor concentrations and efficacy. We provide recommendations for future studies in which pazopanib concentrations are measured.

**Abstract:**

There is a lack of understanding whether plasma levels of anticancer drugs (such as pazopanib) correlate with intra-tumoral levels and whether the plasma compartment is the best surrogate for pharmacokinetic and pharmacodynamic evaluation. Therefore, we aimed to quantify pazopanib concentrations in tumor tissue, to assess the correlation between tumor concentrations and plasma concentrations and between tumor concentrations and efficacy. In this clinical trial, non-metastatic STS patients were treated with neo-adjuvant concurrent radiotherapy and pazopanib. Plasma samples and tumor biopsies were collected, and pazopanib concentrations were measured using liquid chromatography-tandem mass spectrometry. Twenty-four evaluable patients were included. The median pazopanib tumor concentration was 19.2 µg/g (range 0.149–200 µg/g). A modest correlation was found between tumor concentrations and plasma levels of pazopanib (*ρ* = 0.41, *p* = 0.049). No correlation was found between tumor concentrations and percentage of viable tumor cells (*p* > 0.05); however, a trend towards less viable tumor cells in patients with high pazopanib concentrations in tumor tissue was observed in a categorical analysis. Possible explanations for the lack of correlation might be heterogeneity of the tumors and timing of the biopsy procedure.

## 1. Introduction

Pazopanib is a tyrosine kinase inhibitor (TKI) which targets the vascular endothelial growth factor receptor (VEGFR)-1, -2 and -3; platelet-derived growth factor (PDGFR)-α and -β; fibroblast growth factor receptor (FGFR)-1, -3 and -4; and stem cell factor receptor (c-KIT) [1,2]. By targeting these receptors, pazopanib inhibits tumor proliferation and angiogenesis [3]. It is approved for the first-line treatment of advanced or metastatic renal cell carcinoma (RCC) and as second-line therapy of metastatic soft-tissue sarcoma (STS) [1]. Haas et al. showed that the neoadjuvant use of pazopanib 800 mg once daily in combination with 50 Gy preoperative radiotherapy (RT) seems tolerable and shows promising results in terms of efficacy also in patients with non-metastatic, intermediate or high-grade STS of the extremities, in the phase I PASART-1 study (NCT01985295) [4]. The combined treatment regimen was subsequently assessed in the phase II PASART-2 trial (NCT02575066) [5]. The main efficacy endpoint of this single arm trial was the rate of pathological complete response (pCR), defined as ≤5% viable tumor cells in the resection specimen at central pathology review. The combination treatment led to a relatively low rate of major wound complications (24%). Out of 25 patients, 17 patients experienced grade 3 or higher acute toxicities, but as the vast majority of those were asymptomatic, transient ALT/AST elevations or hypertension, the regimen was deemed tolerable. A promising pCR rate of 20% (5/25 patients) was observed. This manuscript reports the results of the pharmacokinetic sub study of the PASART-2 trial.

Pazopanib targets receptors on tumor cells and on cells in the tumor micro-environment (TME). Therefore, at least theoretically, in order for pazopanib to be effective, adequate plasma levels must be achieved, so sufficient pazopanib molecules could potentially arrive in the vicinity of the tumor. Indeed, pazopanib efficacy has been related to plasma trough levels (*C_min_*) in advanced or metastatic RCC, with a significant decrease in tumor size and longer progression free survival (PFS) for patients with a *C_min_* of ≥20.5 mg/L [6,7,8]. In metastatic STS patients with a pazopanib *C_min_* > 20 mg/L, a trend was found towards a decrease in tumor size and better PFS [8,9].

However, multiple factors, such as tumor vascularization, hypoxia and the presence of drug efflux transporters, could influence drug penetration from plasma to tumor tissue [10]. Therefore, drug concentrations in tumor tissue could potentially serve as an even stronger prognostic biomarker, as pazopanib ultimately exerts its effects in the tumor tissue and tumor microenvironment, not in the plasma. Previously published research showed that the drug concentrations of multiple TKIs, for example gefitinib and erlotinib, could be measured in human tumor tissue [11,12,13]. However, these concentrations have not been correlated to efficacy parameters. For pazopanib, a method to measure drug concentrations and distribution in mouse tumor models has been described, which was used to study suboptimal pharmacokinetics as an explanation for drug resistance [14].

In this pharmacokinetic sub-study of the PASART-2 trial, plasma samples and tumor biopsies were taken from patients with non-metastatic STS of the extremities, trunk, chest wall or head and neck region treated with neoadjuvant pazopanib in combination with preoperative RT. The aim of this study was to quantify pazopanib concentrations in tumor tissue and to determine the correlation between tumor and plasma concentrations and between tumor concentrations and efficacy. We hypothesized that pazopanib tumor concentrations would be higher in patients showing response to the treatment.

## 2. Materials and Methods

### 2.1. Study Design

This pharmacokinetic study was embedded in an international, multicenter phase II trial (NCT02575066) [5]. Written informed consent was obtained, the study protocol was approved by the local ethics commissions and the study was conducted in accordance with the declaration of Helsinki.

### 2.2. Study Population

Patients were eligible if they were ≥18 years, had a WHO performance status of ≤1 and a histologically confirmed non-metastatic soft tissue sarcoma localized in the extremities, trunk, chest wall or the head and neck region, for which standard therapy is surgery and radiotherapy. This means the tumor had to be deep-seated and/or >5 cm and/or grade II/III according to the Fédération Nationale des Centres de Lutte Contre Le Cancer (FNCLCC) definition and/or close resection margins had to be anticipated. Patients were excluded if they had prior malignancies (except if they were disease-free for at least 5 years) or recurrent sarcomas and if they received chemotherapy or radiation therapy within 2 weeks prior to first dose of study medication or biological therapy within 28 days or five half-lives. Furthermore, female patients who were pregnant or breast-feeding were excluded, and all patients were required to employ effective methods of birth control.

### 2.3. Study Treatment

The planned radiotherapy dose was 50 Gy in once daily 2 Gy fractions for 5 days a week, from day 1 to day 33. Pazopanib once daily 800 mg was commenced one week prior to the start of radiotherapy on day -7 and continued until radiotherapy completion. Surgery was performed 4–8 weeks post pazopanib and radiotherapy treatment.

### 2.4. Pharmacokinetics

Blood samples were collected in K_2_ EDTA tubes at day 1 and day 22. The samples were centrifuged at 2200× *g* for 10 min. Plasma was stored at −20 °C until analysis.

Tumor samples were collected at day 22 of the study. These samples were snap frozen immediately after the biopsy procedure by immersing them in liquid nitrogen for 30 s. The samples were stored at −80 °C until further processing. Before processing, the tumor samples were weighted, and 300 µL control human K_2_EDTA plasma was added. Subsequently, the samples were homogenized with a Polytron^®^ PT 1200B homogenizer (Kinematica AG) for at least 3 min per sample. Lastly, samples were centrifuged at 3000× *g* for 1 min to get rid of the foam that arose during homogenization. The homogenized samples were stored at −80 °C until analysis.

Concentrations of pazopanib in plasma were measured using a validated liquid chromatography-tandem mass spectrometry (LC-MS/MS) method [15]. The method for measurement of pazopanib in tumor homogenates was based on a validated LC-MS/MS method in plasma; however, the sample preparation was adjusted to concentrate the samples [16]. A 100 µL aliquot of the homogenate was transferred to an Eppendorf tube, and 200 µL of methanol containing 0.1 µg/mL internal standard (^13^C^2^H_3_-pazopanib) was added before the sample was vortex mixed. Subsequently, the sample was centrifuged at 23,100× *g* for 5 min, and 100 µL of the clear supernatant was transferred to a clean reaction tube and mixed with 100 µL of 10 mmol/L ammonium hydroxide in water before injection. Pazopanib could be measured in tumor homogenates in a range of 0.01 to 10 µg/mL.

An experiment was conducted to determine if all pazopanib was released from the tumor sample because for some samples, a small pellet of tumor tissue resided in the Eppendorf tube after homogenization and centrifugation. First, the concentration of pazopanib was measured in the tumor homogenate. Next, the residue was taken, and 200 µL control human K_2_EDTA plasma was added. The sample was homogenized and centrifuged again, following the same method as described earlier. The concentration in the plasma supernatant was measured. After that, the procedure was repeated, and the concentration in the plasma supernatant was measured.

**Figure 1:**
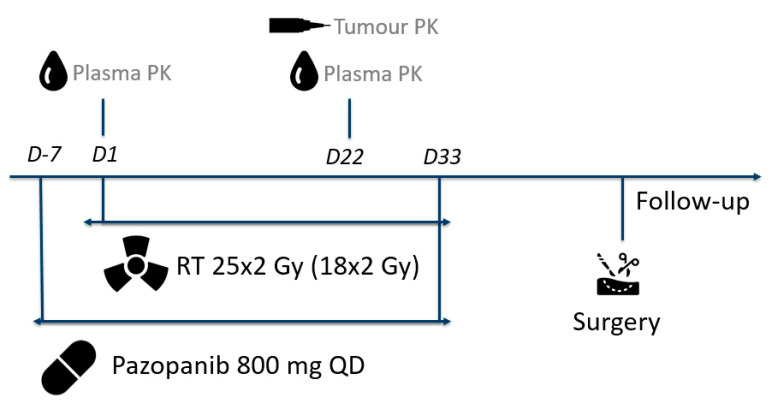
Overview of study procedures for the PASART-II pharmacokinetic study.

The pazopanib plasma concentrations were corrected for the time after dose at which the sample was obtained by calculating pazopanib trough levels. The following formula was used [17]:

$$C_{min}=C_{measured}\times 0.5^{\frac{\left(dosing\;interval-TAD\right)}{t\frac{1}{2}}}$$

where *C_min_* is the calculated pazopanib trough level, *C_measured_* is the measured pazopanib concentration, TAD is the time after dose, and t½ is the elimination half-life, which is 31 h for pazopanib [1]. The pazopanib tumor concentrations were corrected for the dilution in human plasma and the weight of the tumor sample, resulting in a concentration pazopanib in mg/g tumor tissue. In Figure 1, an overview of study procedures is given.

### 2.5. Efficacy Endpoint

For this pharmacokinetic study, the percentage of viable tumor cells estimated on representative slides of each individual surgical specimen under the light microscope, as reported by an experienced soft tissue tumor pathologist (JVMGB), was used as efficacy parameter. Additionally, the percentage of viable tumor cells in the tumor biopsy on day 22 was scored in the same way and compared with the results from the surgical specimen.

### 2.6. Statistics

Statistical analyses were performed using R version 3.6.3 (R Project, Vienna, Austria). Descriptive statistics were used to summarize treatment outcomes and pazopanib plasma and tumor levels. The Wilcoxon signed rank test was employed to test for differences between pazopanib plasma levels on day 1 and day 22. To study the associations between pazopanib plasma levels and pazopanib tumor levels as well as the percentage of viable tumor cells in tumor tissue on day 22 and in the resection specimen, Spearman’s rank correlation was used. The associations between pazopanib tumor levels and percentage of viable tumor cells, as well as pazopanib plasma trough levels and percentage viable tumor cells, were studied both numerically, assuming a linear association and categorically, assuming a threshold for efficacy. For the numerical analysis of the association between drug concentration and efficacy, the Spearman’s rank correlation was used. For the categorical analysis, pazopanib tumor levels were divided in quartiles, and the association was tested with the Kruskal−Wallis test. Secondly, pazopanib tumor levels were divided in two equal groups, and the association was tested with the Wilcoxon signed rank test. The pazopanib plasma trough levels were divided in groups with a low and adequate exposure, according to the threshold value of 20 mg/L [6], and the association was tested with the Wilcoxon signed rank test. *p* ≤ 0.05 was considered statistically significant.

## 3. Results

### 3.1. Patient Characteristics

Between March 2016 and December 2018, 25 patients with non-metastatic soft tissue sarcoma were included in the study. The first 21 patients received 50 Gy radiotherapy. After an interim analysis, the protocol was amended, and four additional patients were added to the study, who were treated with a lower dose of 36 Gy radiotherapy in once daily 2 Gy fractions, for 5 days a week from day 1 to day 24. For one patient, we were not able to adequately homogenize the tumor tissue. Therefore, this patient was excluded from the analysis. Patient characteristics of the 24 remaining evaluable patients are shown in Table 1. Histological subtyping was done by the World Health Organization (WHO) 2013 classification [18].

### 3.2. Pharmacokinetics

In order to determine if pazopanib was released from the tumor sample, a recovery experiment was conducted. Almost all pazopanib was released from the tumor sample in this experiment. In the plasma supernatant, after the first extra homogenization step of the residue, only 5% of pazopanib was measured compared to the tumor homogenate. After the second homogenization step, less than 2% of pazopanib was measured compared to the tumor homogenate (value under lower limit of quantification (LLOQ)).

Pazopanib plasma concentrations were measured on day 1 and day 22 of the study, with the exception of two patients where plasma concentrations were measured on day 1 or day 22 only. The median pazopanib trough concentration in plasma was 37.1 mg/L (range 4.47–78.1 mg/L) on day 1 and 33.5 mg/L (range 8.38–120.7 mg/L) on day 22. There was no statistically significant difference in plasma trough levels between day 1 and day 22 (*p* = 0.64), as shown in Figure S1. For further data analysis, plasma trough levels on day 22 were used as also tumor concentrations were measured on day 22. For a single patient for whom no plasma trough level was available on day 22, the plasma trough level on day 1 was used.

**Figure 2:**
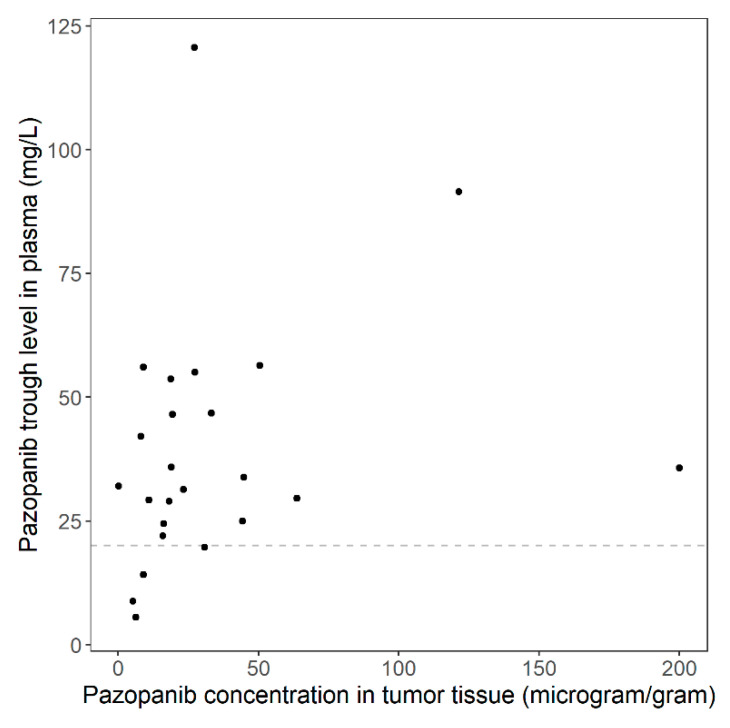
Pazopanib concentrations in tumor tissue versus pazopanib trough levels in plasma (ρ = 0.41, *p* = 0.049). The dashed line represents a pazopanib trough level of 20 mg/L, which is advised as the target for adequate drug exposure.

The median pazopanib tumor concentration measured in tumor homogenates taken on day 22 was 19.2 µg/g (range 0.149–200 µg/g). Two patients had high pazopanib tumor concentrations compared to the other patients (200 and 121 µg/g). One of these patients also had a high pazopanib plasma concentration (plasma concentrations were 91 and 36 mg/L, respectively, for the two patients). Both patients did not experience grade 3 or 4 toxicity. In addition, one patient was found to have a very low pazopanib tumor concentration (0.149 µg/g). This patient had an adequate pazopanib plasma concentration (32 mg/L) and experienced grade 3 or 4 toxicity, including hypertension. Overall, a modest correlation was found between tumor concentrations and plasma trough levels of pazopanib (*ρ* = 0.41), which was borderline significant (*p* = 0.049); see Figure 2.

For 18 patients, the percentage of viable tumor cells could be evaluated on the biopsy from day 22. No statistically significant correlation was found between the percentage of viable tumor cells in the tumor biopsy on day 22 and in the resection specimen (*ρ* = 0.21; *p* = 0.37); see Figure S2. For further data analysis, percentage of viable tumor cells in the resection specimen was used, in order to study tumor concentrations as a biomarker for efficacy of the total treatment.

**Figure 3:**
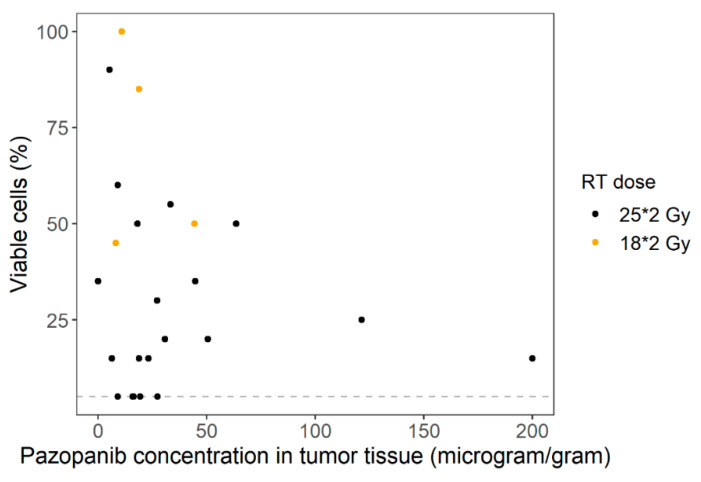
Pazopanib concentrations in tumor tissue versus percentage of viable tumor cells in resection specimen (ρ = −0.10, *p* = 0.63). The dashed line represents the cut-off value for a good response, which is ≤5% viable tumor cells. Patients which are shown as black dots were treated with a radiotherapy dose of 50 Gy, and patients which are shown as orange dots were treated with a radiotherapy dose of 36 Gy.

**Figure 4:**
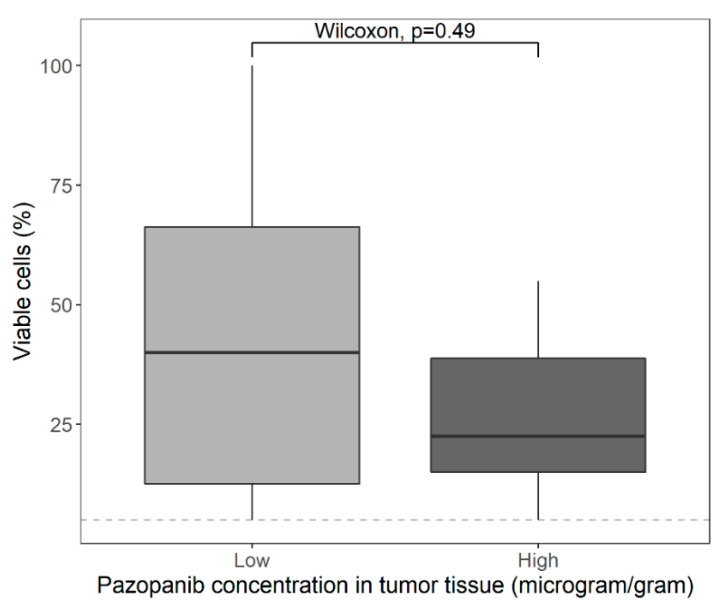
Pazopanib concentrations in tumor tissue, equally divided in two groups, versus percentage of viable tumor cells in resection specimen. The dashed line represents the cut-off value for a good response, which is ≤5% viable tumor cells.

No statistically significant correlation was found between tumor concentrations and efficacy measured by percentage of viable tumor cells in the resection specimen when numerically studied (*ρ* = −0.10; *p* = 0.63), as depicted in Figure 3. When categorically analyzed, no statistically significant difference was found either (*p* = 0.49 when divided in two groups; *p* = 0.67 when divided in quartiles). However, visual inspection indicates a trend towards less viable tumor cells in patients with high pazopanib concentrations in tumor tissue, as shown in the boxplots in Figure 4 (median 40.0% viable tumor cells for low tumor concentrations, and 22.5% for high tumor concentrations) and Figure S3.

Furthermore, no clear pattern was observed of the effect of radiotherapy dose and tumor type, as shown in Figure 3 and Figure S4, respectively.

No statistically significant correlation was found between pazopanib plasma trough levels and percentage of viable tumor cells in resection specimen either (*ρ* = 0.11, *p* = 0.62), as shown in Figures S5 and S6 (with radiotherapy and tumor type included in the plot, respectively). In a categorical analysis, a non-significant (*p* = 0.67) trend towards more viable tumor cells in patients with adequate pazopanib trough levels in plasma (>20 mg/L) was observed (Figure S7). However, this analysis was only based on four patients with a low exposure (<20 mg/L).

## 4. Discussion

In medical oncology, for several drugs it has been shown that drug levels in plasma can be measured to guide patient dosing for efficacy and to assess toxicity [19]. Studies assessing intra-tumoral drug levels are extremely rare, and to the best of our knowledge, for TKIs only erlotinib and gefitinib have been measured in human tumor tissue [11,12]. Our study is the first to show that pazopanib levels can be quantified both in plasma and in human sarcoma tumor tissue. It is also the first to assess the correlation of intra-tumoral TKI drug levels with efficacy. A modest correlation was found between tumor concentration and plasma concentration (*ρ* = 0.41, *p* = 0.049). No statistically significant correlation between tumor concentration and percentage viable tumor cells was found, both when tested numerically assuming a linear relationship and categorically assuming a threshold for efficacy (numerically tested: *ρ* = −0.10, *p* = 0.63; categorically tested: *p* = 0.49).

Only a modest correlation was found between tumor and plasma concentrations. Therefore, tumor concentrations can provide additional information about distribution and efficacy of pazopanib. Besides, a reason for the modest correlation could also be that in all except four patients, a *C_min_* > 20 mg/L was measured. The previously reported relationship between *C_min_* and clinical outcomes suggests the presence of a minimal effective concentration pazopanib in the tumors above this value [6,7,8,9].

No statistically significant correlation between tumor concentrations and efficacy was found, but categorical analysis of the data indicates a trend towards less viable tumor cells in patients with high pazopanib concentrations in tumor tissue. We cannot draw strong conclusions based on our research; however, the following factors could have contributed to our findings.

First of all, the timing of the tumor biopsy procedure, which was performed on day 22 in our study (4 weeks after start of pazopanib and 3 weeks after start of radiotherapy), could have affected the results. In order to have an anti-tumor effect, pazopanib should be taken up by tumor tissue. This uptake is expected to be dependent on more factors than only pazopanib plasma levels. One of these factors could be the effect of pazopanib itself. Historically, anti-angiogenics such as pazopanib were expected to cause tumor vessel death and regression, thereby depriving the tumor of oxygen and nutrients needed for rapid cell proliferation and tumor growth. However, multiple studies have suggested that anti-angiogenic treatment might actually initially lead to enhanced tumor perfusion and oxygenation [20,21,22]. In many tumor micro-environments, there is an overexpression of pro-angiogenic factors such as VEGF. This leads to new vessel recruitment to the tumor but also causes a chaotic vascular network with often leaky vessels [23,24]. Anti-angiogenics normalize the balance of pro- and anti-angiogenic factors and thereby lead to a better-organized vascular network and more adequate vascular pericyte coverage [25,26]. This explains the possible synergism when combining anti-angiogenic treatment with radiotherapy or chemotherapy [27,28], as the latter treatment modalities are known to be more effective in well-perfused, oxygenated tumors. The vessel normalization effect is thought to be temporary and can be described by a ‘normalization window’ [29,30,31]. With sustained anti-angiogenic treatment, subsequently the balance shifts towards more anti-angiogenic factors, which will lead to regression of blood vessels [25]. Therefore, the measured tumor concentrations of pazopanib could be affected by both the normalization of the tumor vasculature, leading to improved distribution of pazopanib to tumor tissue, or by vessel regression, through which pazopanib could limit its own distribution to the tumor. Additionally, the moment of evaluating tumor response might also influence the results. Four weeks (day 7 until day 22) could be too short for pazopanib to already have a significant effect on the viability of tumor cells. No statistically significant correlation was found between the percentage of viable tumor cells in tumor tissue on day 22 and in the resection specimen (neo-adjuvant treatment until day 33, 4–8 weeks later surgery was performed). In Figure S2, it can be seen that 10 out of 18 patients had 100% viable tumor cells in the tumor at day 22, while only one patient had 100% viable tumor cells in the resection specimen, which suggests that day 22 may be too early to estimate response. Therefore, it might be better to evaluate treatment efficacy at a later time point. In the study in which a correlation between pazopanib plasma concentrations of ≥20.5 mg/L and efficacy was found, pazopanib plasma concentrations were also measured after 4 weeks, and tumor response was evaluated 8 weeks thereafter [6]. This supports the choice to evaluate response in the resection specimen for the tumor concentration-response analysis.

A second factor that could play a role in explaining the observed results is heterogeneity, both between tumor types and within the tumor. In our study, a wide variety of STS tumor subtypes was included. A difference between tumor types is for example the vasculature of the tumor [32,33]. As described before, tumor vasculature could influence the distribution of pazopanib to the tumor. Next to intra-tumoral uptake of pazopanib, intra-tumoral distribution is crucial for the effect of pazopanib [14]. Torok et al. described that intra-tumoral distribution of pazopanib was inhomogeneous with most pazopanib distributed to necrotic areas [14]. We took one image-guided biopsy (16G) per patient. Although the use of imaging ensures that the biopsy is not taken in a necrotic tumor area, the biopsy might not be representative for the whole tumor because of the mentioned inhomogeneity.

Other factors affecting our results could firstly be the concurrent radiotherapy. The effects of the neo-adjuvant therapy we observed in the resection specimen of the patients could also predominantly be due to the effect of radiotherapy. Therefore, the pazopanib tumor tissue concentrations could have been of secondary importance. Furthermore, radiotherapy also affects the tumor vasculature [34]. Secondly, the small number of patients and few responses in the patient population (five patients achieving a pathological complete response) could also be a reason for the lack of a correlation between pazopanib plasma levels and efficacy. This absence of correlation for systemic pazopanib concentrations could, in turn, explain the lack of a correlation between pazopanib tumor concentrations and efficacy.

Lastly, the homogenization procedure and bioanalytical method to measure pazopanib in tumor tissue were not validated. Theoretically, it could be possible that not all tumor cells were broken down by the homogenization procedure and that thereby, not all pazopanib was released from the tumor tissue. This could have influenced the studied correlation between tumor and plasma concentrations and between tumor concentrations and efficacy. However, we conducted a recovery experiment in which tumor concentrations of pazopanib in the residues were only 5% and <2% compared to the concentration in the tumor homogenate before extra homogenization steps. These findings support the reliability of the used methods.

To avoid some of the limitations we encountered in the current study design in future studies, the following suggestions are proposed in case tumor concentrations of anti-angiogenic drugs are measured: (1) measure drug tumor concentrations at different time points, to be able to study the changes over time, or perform a preclinical study to determine the optimal time for the biopsy procedure; (2) account for tumor heterogeneity and increase the chance that the biopsy of the tumor is representative (e.g., through combination with imaging-based analysis of drug distribution).

## 5. Conclusions

Measurement of pazopanib concentrations in human tumor tissue is possible. A modest correlation between tumor and plasma concentrations was found as well as no statistically significant correlation between tumor concentrations and percentage of viable tumor cells. In a categorical analysis, no statistically significant relationship was found either; however, we found a trend towards less viable tumor cells in the 50% of patients with the highest pazopanib tumor tissue concentrations. The non-significant relationship between tumor concentrations and efficacy could possibly be explained by heterogeneity of the tumors and timing of biopsies. For future studies, in which tumor concentrations of anti-angiogenic drugs are measured, it is suggested to measure drug tumor concentrations at different time points or to base the timing of the biopsy procedure on preclinical research and to account for tumor heterogeneity in the biopsy procedure.

## Figures and Tables

**Table 1:** Patient characteristics (*n* = 24).

Characteristic	*n* (%) or Median (Range)
**Gender**	
Male	14 (58)
Female	10 (42)
**Age (years)**	57 (24–79)
**Tumor histology**	
Undifferentiated pleomorphic sarcoma	9 (38)
Myxofibrosarcoma	8 (33)
Spindle cell sarcoma (not otherwise specified)	2 (8)
Myxoid liposarcoma	1 (4)
Synovial sarcoma	1 (4)
Spindle cell rhabdomyosarcoma	1 (4)
Clear cell sarcoma	1 (4)
Malignant peripheral nerve sheath tumor	1 (4)
**Pazopanib trough levels in plasma on day 22 (mg/L)**	34.8 (8.38–120.7)
**Pazopanib concentrations in tumor tissue (µg/g)**	
Total	19.2 (0.149–200)
Undifferentiated pleomorphic sarcoma	27.4 (0.149–121)
Myxofibrosarcoma	18.9 (5.31–200)
Spindle cell sarcoma (not otherwise specified)	36.4 (9.08–63.7)
Myxoid liposarcoma	16.3 (N/A)
Synovial sarcoma	27.3 (N/A)
Spindle cell rhabdomyosarcoma	18.2 (N/A)
Clear cell sarcoma	16.0 (N/A)
Malignant peripheral nerve sheath tumor	11.0 (N/A)

## Data Availability

The data that support the findings of this study are available from the corresponding author upon reasonable request.

## Supplementary Materials

The following are available online at https://www.mdpi.com/article/10.3390/cancers13225780/s1. Figure S1: Pazopanib trough levels in plasma on day 1 versus day 22. Figure S2: Percentage of viable tumor cells in tumor tissue on day 22 (D22) versus in resection specimen. Figure S3: Pazopanib concentrations in tumor tissue, divided in quartiles, versus percentage viable tumor cells in resection specimen. Figure S4: Pazopanib concentrations in tumor tissue versus percentage viable tumor cells in resection specimen (ρ = −0.10, *p* = 0.63). Figure S5: Pazopanib trough levels in plasma versus percentage viable tumor cells in resection specimen (ρ = 0.11, *p* = 0.62). Figure S6: Pazopanib trough levels in plasma versus percentage viable tumor cells in resection specimen (ρ = 0.11, *p* = 0.62). Figure S7: Pazopanib trough levels in plasma, divided in a low and high exposure using a cut-off value of 20 mg/L, versus percentage viable tumor cells in resection specimen.
