# Supplementary material for: Intra-Tumoral Pharmacokinetics of Pazopanib in Combination with Radiotherapy in Patients with Non-Metastatic Soft-Tissue Sarcoma
Source: Cancers (Basel). 2021 Nov 18;13(22):5780. doi: 10.3390/cancers13225780 (PMC8616484; doi:10.3390/cancers13225780)
Supplement: Supplementary file 1 [file cancers-13-05780-s001.zip › cancers-1432577-supplementary.pdf]

Supplementary Material

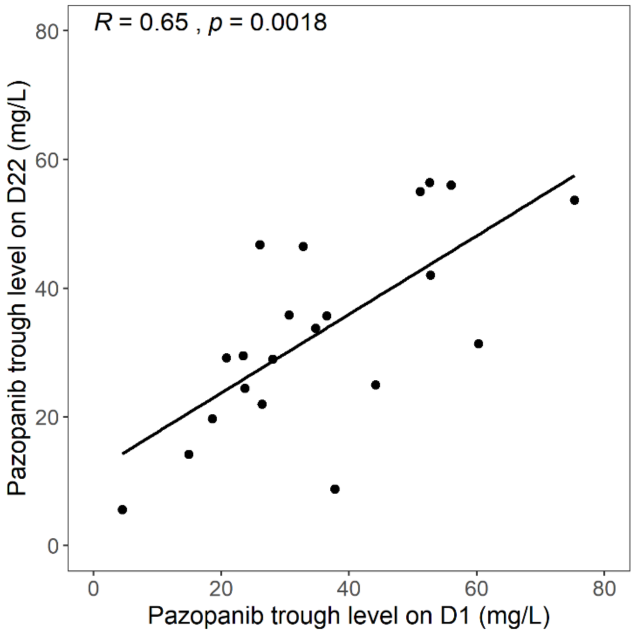

Figure S1: Pazopanib trough levels in plasma on day 1 versus day 22. The black line represents the linear trend in the observations. D1, day 1; D22, day 22

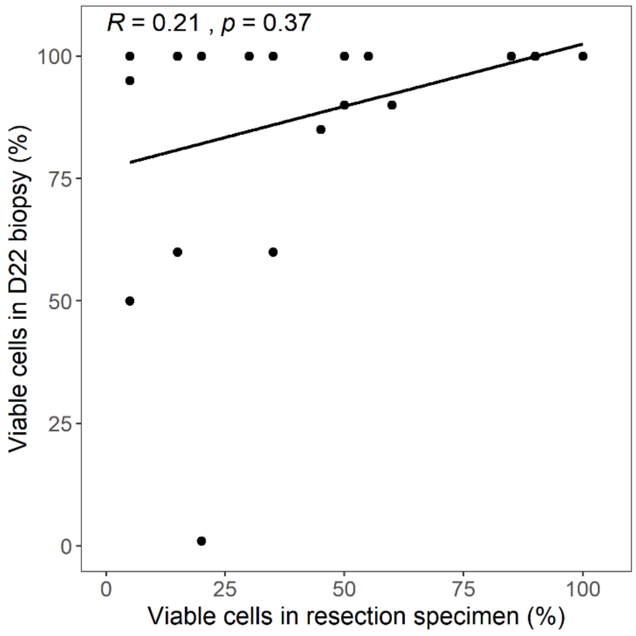

Figure S2: Percentage of viable tumor cells in tumor tissue on day 22 (D22) versus in resection specimen. The black line represents the linear trend in the observations. D22, day 22

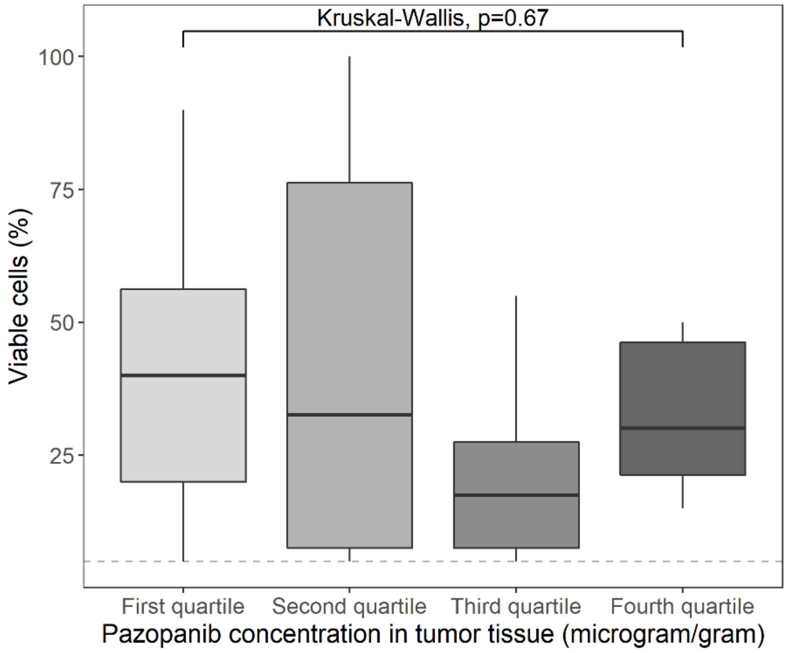

Figure S3: Pazopanib concentrations in tumor tissue, divided in quartiles, versus percentage viable tumor cells in resection specimen. The dashed line represents the cut-off value for a good response, which is ≤5% viable tumor cells.

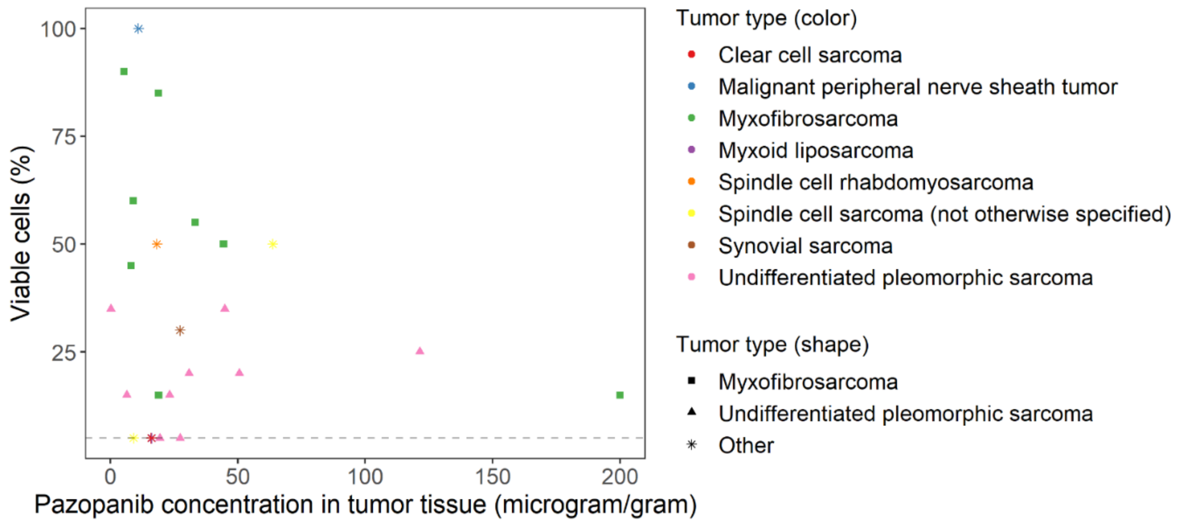

Figure S4: Pazopanib concentrations in tumor tissue versus percentage viable tumor cells in resection specimen (ρ=-0.10, p=0.63). The dashed line represents the cut-off value for a good response, which is ≤5% viable tumor cells. The color and shape of the symbols corresponds with tumor type.

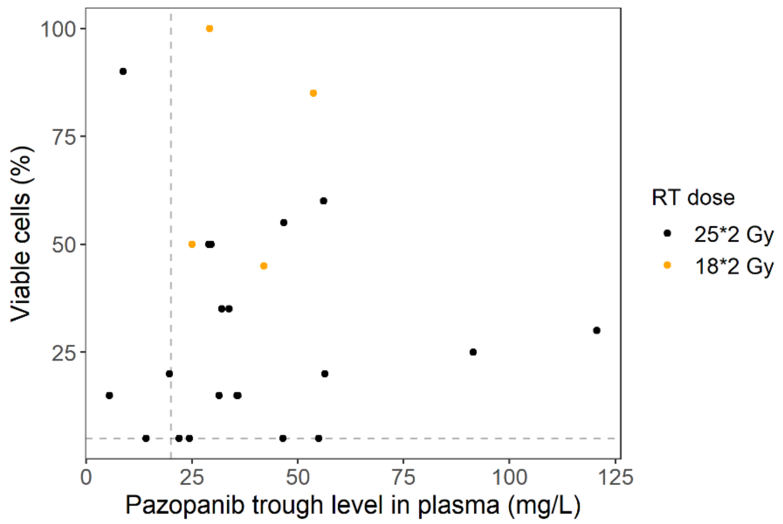

Figure S5: Pazopanib trough levels in plasma versus percentage viable tumor cells in resection specimen (ρ=0.11, p=0.62). The horizontal dashed line represents the cut-off value for a good response, which is ≤5% viable tumor cells. The vertical dashed line represents a pazopanib trough level of 20 mg/L, which is advised as the target for adequate drug exposure. Patients which are shown as black dots were treated with a radiotherapy dose of 50 Gy, patients which are shown as orange dots were treated with a radiotherapy dose of 36 Gy.

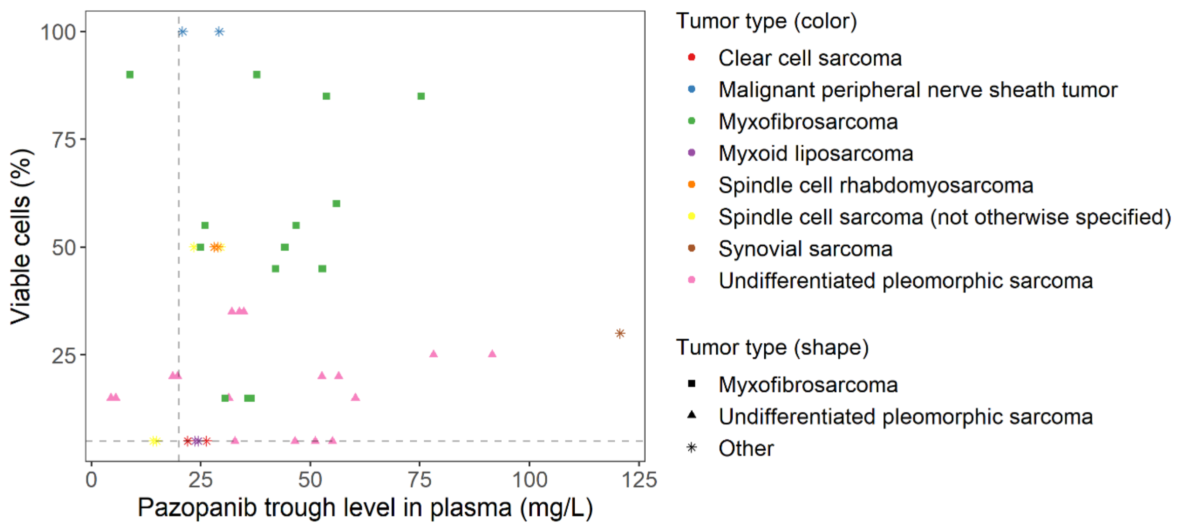

Figure S6: Pazopanib trough levels in plasma versus percentage viable tumor cells in resection specimen (ρ=0.11, p=0.62). The horizontal dashed line represents the cut-off value for a good response, which is ≤5% viable tumor cells. The vertical dashed line represents a pazopanib trough level of 20 mg/L, which is advised as the target for adequate drug exposure. The color and shape of the symbols corresponds with tumor type.

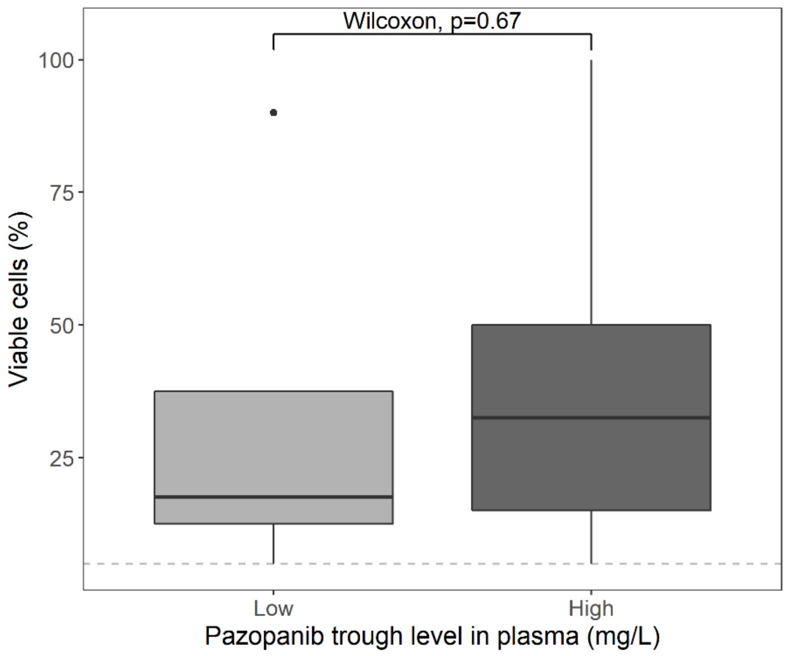

Figure S7: Pazopanib trough levels in plasma, divided in a low and high exposure using a cut-off value of 20 mg/L, versus percentage viable tumor cells in resection specimen. The dashed line represents the cut-off value for a good response, which is ≤5% viable tumor cells.
